# GENERATING VIDEO VISITS FOR RURAL OLDER VETERANS IN TELE-GERIATRIC MENTAL HEALTH SERVICES

**DOI:** 10.1093/geroni/igae098.2522

**Published:** 2024-12-31

**Authors:** Christine Gould, Chalise Carlson, Marika Humber, Amanda Peeples, Zachary Burningham, Ranak Trivedi

**Affiliations:** VA Palo Alto Healthcare System, Palo Alto, California, United States; Department of Veterans Affairs, Palo Alto, California, United States; VA Palo Alto Healthcare System, Palo Alto, California, United States; Corporal Michael J. Crescenz Department of Veterans Affairs Medical Center, Philadelphia, Pennsylvania, United States; VA Salt Lake City Healthcare System, Salt Lake City, Utah, United States; Stanford University/VA Palo Alto Healthcare System, Palo Alto, California, United States

## Abstract

Telehealth services increase access to specialty mental health services for rural older adults. Within the Veterans Health Administration (VHA), older Veterans are more likely to receive audio-only mental telehealth services compared with younger Veterans. Using a mixed methods approach, we examined the use of video telehealth services by geriatric mental health (GMH) providers in four regional VHA telehealth hubs. Between October 2022 and September 2023, 351 rural Veterans with a mean age of 75.1 years (SD = 8.8) received services from one or more GMH provider. Patients were mostly white (88.6%), males (95.4%) with high care needs (CAN = 81.6 out of 100, indicating highest need). Of the 1,440 encounters delivered, 49% were video to home, 27% were video to clinic, 19% were telephone, and 5% were e-consultation services. Through interviews with GMH providers, we identified technology-related barriers and solutions used to help rural Veterans be seen via video. Findings suggested that barriers to video services included lack of home internet and/or devices, poor connection, and audio or video issues. Solutions included using VHA resources to obtain iPads for patients who lacked connectivity/devices and prioritizing getting patients into local clinic when needed for video visits. Providers described utilizing the telehealth clinical technician to help overcome patient barriers and discomfort with digital technology through teaching patients and caregivers to use home telehealth software. With troubleshooting, use of resources and training, high quality video telehealth services can be delivered successfully to older rural Veterans and increase access to GMH care.

